# Treatment of Human Placental Choriocarcinoma Cells with Formaldehyde and Benzene Induced Growth and Epithelial Mesenchymal Transition via Induction of an Antioxidant Effect

**DOI:** 10.3390/ijerph14080854

**Published:** 2017-07-29

**Authors:** Hae-Miru Lee, Soo-Min Kim, Kyung-Chul Choi

**Affiliations:** Laboratory of Biochemistry and Immunology, College of Veterinary Medicine, Chungbuk National University, Cheongju, Chungbuk 28644, Korea; adekato8898@gmail.com (H.-M.L.); tnals1613@gmail.com (S.-M.K.)

**Keywords:** cigarette smoke, formaldehyde, benzene, placenta choriocarcinoma, cell cycle, EMT

## Abstract

Cigarette smoke (CS) causes about 480,000 deaths each year worldwide, and it is well-known to have harmful effects on the human body, leading to heart disease, stroke, lung cancer, and cardiovascular problems. In this study, the effects of formaldehyde (FA) and benzene (Bz), the main components of CS, on cell proliferation and epithelial mesenchymal transition (EMT) of JEG-3 human choriocarcinoma cells were examined to confirm the relationship between CS components and placenta carcinoma. Upon MTT assay, FA (10^−8^ M to 10^−5^ M) and Bz (10^−11^ M to 10^−8^ M) increased JEG-3 cell proliferation. Western blot assay revealed that the protein expression of cyclin D1 and E1 increased, while the levels of p21 and p27 were reduced following treatment. In Scratch assay, FA (10^−8^ M and 10^−5^ M) and Bz (10^−11^ M and 10^−8^ M) increased migration of JEG-3 cells at 24 h and 48 h compared with that at 0 h. In addition, the expression of the epithelial marker, E-cadherin, was significantly decreased, while the expression of the mesenchymal marker, N-cadherin, was significantly increased by FA (10^−8^ M and 10^−5^ M) and Bz (10^−11^ M and 10^−8^ M). snail and slug transcriptional factors were associated with EMT, which were also up-regulated by FA and Bz, indicating that FA and Bz lead to an increase in the EMT process in JEG-3 choriocarcinoma cells. We further evaluated reactive oxygen species (ROS) and activation of antioxidant effect using dichlorofluorescin diacetate (DCFH-DA) and Western blot assay. FA and Bz increased the ROS production and an antioxidant related marker, Nrf2, in JEG-3 cells. However, eIF2α levels were reduced by FA and Bz via activation of the antioxidant reaction. Taken together, these results indicated that FA and Bz induce the growth and migration of human choriocarcinoma cells via regulation of the cell cycle and EMT and activation of ROS and antioxidant related markers.

## 1. Introduction

Cigarette smoke (CS) causes about 480,000 deaths each year worldwide, and is well known to be harmful to humans, being responsible for heart disease, stroke, lung cancer, and cardiovascular problems. Moreover, CS is known to be one of the largest risk factors of cancer via a variety of bio-mechanisms [1,2]. In addition, previous studies reported that CS can cause death or survival of different cancer cells at the same time [3,4].

Many previous studies have investigated the relationship between lung cancer and smoking [5], and recent studies have focused on women as the proportion of female smokers has increased worldwide [6]. This causes an increase in female diseases associated with CS. In addition, maternal smoking during pregnancy also influences the health of the fetus and fertility, leading to low birth weight, premature delivery, spontaneous abortion, placental abruption, and ectopic pregnancy [7].

CS generally consists of roughly 5000 different chemicals, among which approximately 150 are toxicants [8]. In a previous study, formaldehyde (FA) and benzene (Bz) were reported to be included in the typical materials belonging to CS [9,10], and CS is also known to be one of the major exposure routes of FA and Bz [11]. Therefore, smokers and secondhand smokers have higher concentrations of FA and Bz in their blood due to higher exposure levels compared to that of the unexposed group or non-smokers [12]. In fact, the outdoor exposure levels of FA and Bz are as low as 1–20 μg/m^3^ and 6 μg/m^3^, respectively, however, the indoor levels of FA and Bz go up to about 120 μg/m^3^ and 10.5 μg/m^3^, respectively, on average when one cigarette is smoked [11].

The Euro and World Health Organization (WHO) reported that FA concentrations range from 60 to 130 mg/m^3^ in CS [13] and people are exposed to about 0.9–2.0 mg when they smoke 20 cigarettes/day; the concentration exposure of Bz was reported to be 1.8 mg/day for a smoker, and 0.05 mg/day for a passive smoker. The international agency for research on cancer (IARC) classified FA and Bz into human carcinogens (group 1). In the present study, the connection between FA and Bz, major CS components, and human choriocarcinoma was investigated to elucidate the harmful effects of CS on the human choriocarcinoma cells JEG-3 and its related mechanism.

Human placenta choriocarcinoma, which is known as a malignant form of gestational trophoblastic disease (GTD), is rarely observed in humans; however, when it occurs, it grows quickly and spreads to the organs from the uterus. Accordingly, human placenta related to pregnancy can develop and lead to specialized fetal trophoblasts; therefore, they play an important role in implantation and development of the maternal-fetal interface [14,15]. Approximately 50% of pregnancies in which choriocarcinoma occurs are associated with a hydatidiform mole and approximately 25% of pregnant women experience miscarriage (spontaneous abortion). Moreover, placenta choriocarcinoma is usually mixed with other types of cancer, forming a type of cancer known as a mixed germ cell tumor.

Epithelial mesenchymal transition (EMT) plays an important role in cancer metastasis and survival [16]. Specifically, EMT plays a key role in the progression of cancer. Approximately 90% of all cancer-related deaths are reported to be associated with tumor metastasis [17,18], and EMT is associated with several major characteristics of cellular progress, including metastasis. During EMT, epithelial cells acquire a mesenchymal cell morphology, which is characterized by the cells changing from a cobblestone-like monolayer with apical basal polarity to a flat morphology. In the absence of polarization, the spindle-shaped mesenchymal cells gain the ability to move. In a previous study, nicotine and cigarette smoke extract (CSE) induced invasion and EMT, as well as changes in the expression of EMT-related proteins such as E-cadherin [19,20]. However, the results of studies of the mechanism responsible for this effect were not clear.

Reactive oxygen species (ROS) are constantly produced from internal metabolism or external exposure [21,22]. Many previous studies have shown that ROS are activated by cigarette smoke or its components [23,24]. In the body, ROS affect various biological mechanisms or functions. Oxidants formed in response to physiological cues play important roles in signaling molecules to regulate process such as cell division, metastasis, apoptosis, autophagy, and stress response [25]. In stress response, endoplasmic reticulum stress (ER stress) is known to be related to cell death or cell survival via its effects on cellular processes [26,27] through the protein kinase RNA-like endoplasmic reticulum kinase (PERK) pathway or the unfolded protein response (UPR) pathway. ER stress is also known to be regulated by ROS [28].

ROS are known to activate the antioxidant enzyme Nrf2 through the PERK pathway [29]. Expression of the nuclear factor erythroid 2 (NFE2)-related factor 2 (Nrf2) plays an important role in antioxidant reactions [30]. Nrf2 is phosphorylated by PERK and then dissociates from the Nrf2/ Kelch-like ECH-associated protein 1 (KEAP1) complex after it enters into the nucleus, and it promotes antioxidant gene expression. Overall, this process leads to resistance to oxidative stress and has a cell-protective effect [30,31].

Based on these biological processes, we specifically investigated the effects of FA and Bz on the proliferation and metastasis of JEG-3 choriocarcinoma cells to provide their roles in the induction of cancer progression via ROS production and antioxidation regulated by Nrf2 when FA and Bz are exposed.

## 2. Materials and Methods

### 2.1. Reagents and Chemicals

Formaldehyde (FA) and benzene (Bz) were purchased from Sigma-Aldrich Corp. (St. Louis, MO, USA). All chemicals were dissolved in 100% dimethyl sulfoxide (DMSO; Junsei Chemical Co., Tokyo, Japan).

### 2.2. Cell Culture and Media

The human placenta choriocarcinoma cell line, JEG-3, was purchased from the Korean Cell Line Bank (KCLB, Seoul, Korea). JEG-3 cells were cultured using Dulbecco’s modified Eagle’s medium (DMEM; HyClone Laboratories Inc., Logan, UT, USA) supplemented with 10% heat-inactivated fetal bovine serum (FBS; HyClone Laboratories Inc.), 2% penicillin G and streptomycin (Cellgro; Mediatech, Inc., Manassas, VA, USA), and 1% HEPES (Invitrogen Life Technologies, Carlsbad, CA, USA) at 37 °C in a humidified atmosphere with 5% CO_2_–95% air as previously described [32].

### 2.3. MTT Assay

JEG-3 cells were seeded at a 5 × 10^3^ cells per well in 96-well plates (SPL Life Science, Seoul, Korea) in a humidified atmosphere of 5% CO_2_ at 37 °C. After the cells were incubated with phenol red-free DMEM containing 5% charcoal dextran fetal bovine serum (CD-FBS) medium for 24 h, they were treated with various concentrations of FA and Bz (FA: 10^−8^ to 10^−5^ M and Bz: 10^−11^ to 10^−8^ M) in phenol red-free DMEM with 5% CD-FBS supplemented with 0.1% DMSO for 9 days. During this period, the media were changed to the same new media every third day. DMSO was used as a vehicle to carry the chemicals to the media. 3-(4-5-dimethylthiazol-2-yl)-2.5-dyphenyltetrazolium bromide (MTT; Sigma-Aldrich) solution was used to confirm increased cell proliferation. Each well of the 96-well plates was treated with 10 μL (5 mg/mL solution) and the plates were incubated for 3 h at 37 °C in a humidified atmosphere of 5% CO_2_. Supernatants were removed and 100 μL DMSO was added to each well to dissolve resultant formazan crystals. Each well was measured using an ELISA reader (VERSA man, Corp., Molecular Devices, Sunnyvale, CA, USA) at an optical density (OD) value of 540 nm and then used to calculate the number of viable cells.

### 2.4. Scratch Assay

JEG-3 cells were cultured to 80% of confluent growth in each well of 6-well plates (SPL Life Science, Seoul, Korea) at 37 °C in a humidified atmosphere of 95% and 5% CO_2_. To perform the scratch assay, a region with the same length and width was scratched, after which JEG-3 cells were treated with negative control (1% DMSO) or formaldehyde (10^−8^ M and 10^−5^ M) and Bz (10^−11^ M and 10^−8^ M) with medium containing 5% CD-FBS, then incubated for 48 h. Next, images were captured with a microscope under 40× magnification at 0 h, 24 h, and 48 h after treatment. The percentage of unrecovered scratched length was measured by dividing the uncovered area at 0 h, 24 h, and 48 h by the initial wound area at time zero. Migration was measured and quantitative analysis was conducted using an Olympus Cellsens dimension program.

### 2.5. Protein Extraction and Western Blot Assay

To measure the protein expression of cyclin D1, cyclin E1, p21, p27, E-cadherin, N-cadherin, snail, slug, and glyceraldehyde 3-phosphate dehydrogenase (GAPDH), 1 × 10^6^ JEG-3 cells were seeded in 100 mm culture dishes (SPL Life Sciences, Corp.), then incubated with FA at a concentration of 10^−8^ M or 10^−5^ M or with Bz at a concentration of 10^−11^ M or 10^−8^ M, respectively, for 72 h. Cells were treated with 0.1% DMSO as a control. Following treatment, whole cell lysates of JEG-3 cells were prepared in 80 μL 1× radioimmunoprecipitation assay(RIPA) buffer (50 mM Tris-HCl; pH 8, 150 mM NaCl, 1% Triton X-100 (Sigma-Aldrich, St. Louis, MO, USA), 0.5% deoxycholic acid (Sigma-Aldrich, St. Louis, MO, USA), and 0.1% SDS). Total protein concentrations were quantified using bicinchoninic acid (BCA; Sigma-Aldrich, Corp.), after which 50 μg of total protein was separated by SDS-polyacrylamide gel electrophoresis (SDS-PAGE). Proteins were then transferred to a polyvinylidene difluoride (PVDF) membrane (BioRad Laboratories, Corp., Hercules, USA) after the membranes were blocked through treatment with 5% bovine serum albumin (BSA; Sigma-Aldrich, Corp, St. Louis, MO, USA) for 90 min at room temperature. The membrane was then incubated overnight at 4 °C with mouse monoclonal anti-GAPDH antibody (Abcam plc, Cambridge, UK), mouse monoclonal anti-cyclin D1 antibody (Abcam plc.), rabbit polyclonal anti-cyclin E1 antibody (Abcam plc.), mouse monoclonal anti-p21 antibody (Abcam plc.), rabbit monoclonal anti-p27 antibody (Abcam plc.), mouse monoclonal anti-N-cadherin antibody (Abcam plc.), mouse monoclonal anti-snail antibody (Cell Signaling Technology, Inc, Danvers, MA, USA), mouse monoclonal anti-slug antibody (Abcam plc.), rabbit monoclonal anti-Nrf2 (Abcam plc.), and rabbit polyclonal anti-phospho-eIF2α antibody (Cell Signaling). Primary antibody binding was detected with horse radish peroxidase (HRP)-conjugated anti-rabbit IgG or anti-mouse IgG (1:2000, Thermo Scientific, Corp, Rockford, IL, USA) for 2 h at room temperature. Target proteins were used with Ez westlumi-plus (ATTO, Corp, Tokyo, Japan) and detected by luminograph II (ATTO, Corp). All protein expression level values were normalized against GAPDH protein.

### 2.6. Determination of ROS Production

Intracellular ROS in JEG-3 cells was observed as previously described using 20,70-dichlorofluorescein diacetate (DCF-DA) (22). JEG-3 cells were seeded at 2 × 10^4^ cells per well in a 96-well plate with phenol-Red free DMEM media containing 5% CD-FBS. After 24 h, the medium was changed to a new medium with FA (10^−8^ M and 10^−5^ M) or Bz (10^−11^ M and 10^−8^ M). After 72 h incubation, the culture medium was removed, and each well was treated with new medium containing 200 µL DCF-DA solution (10 mM-in PBS) for 30 min. This plate was then placed on a shaker for 10 min in a dark room at room temperature, after which the fluorescence was measured using a fluorescence microscope (IX73 fluorescence microscope, Olympus, Japan).

### 2.7. Data Analysis

All experiments were conducted at least three times, and all data were analyzed with the Graph-pad Prism software (San Diego, CA, USA). Data were expressed as the means ± SD and analyzed by one-way analysis of variance (ANOVA) followed by Dunnett’s multiple comparison test. The *p*-values < 0.05 were considered to be statistically significant.

## 3. Results

### 3.1. FA and Bz Induced Increased Cell Proliferation

**Figure 1:**
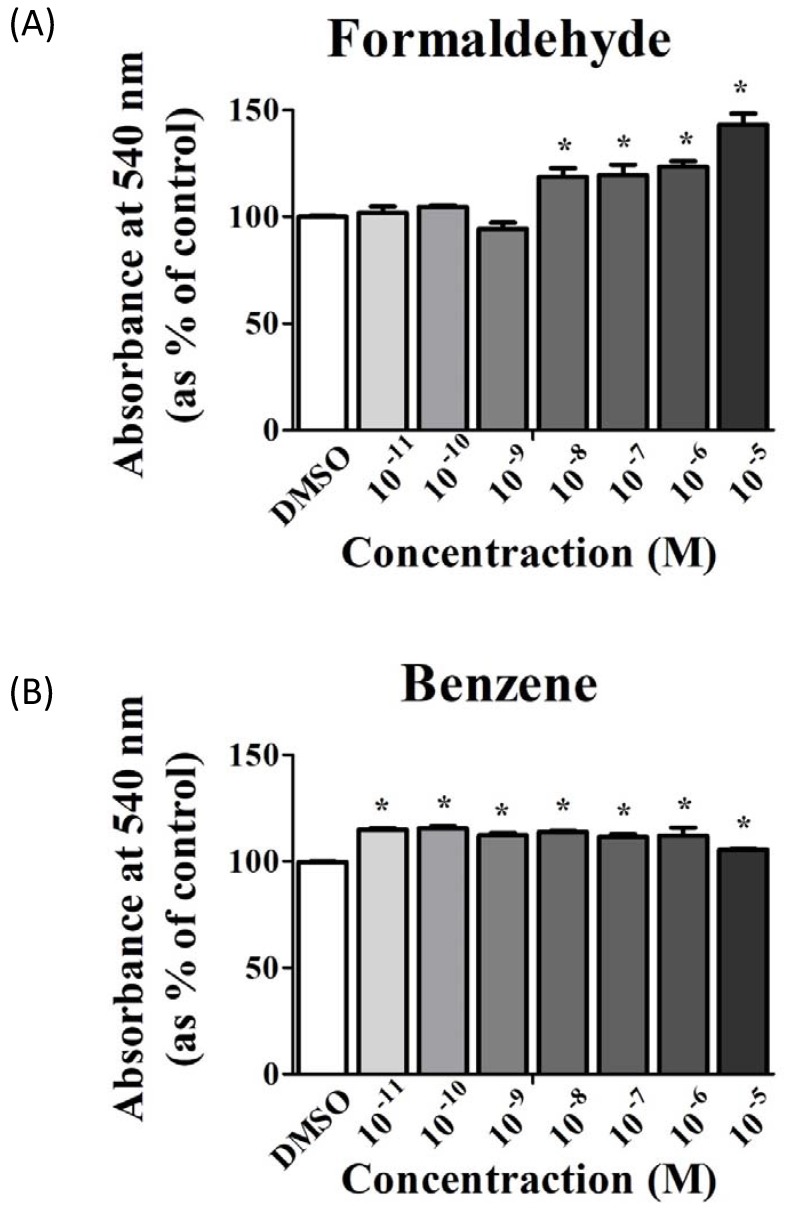
Effect of cigarette smoke components on the proliferation of human placenta carcinoma cells JEG-3. JEG-3 cells were seeded at 5000 cell/well in 96-well plates. After 1 day of incubation, medium treated with (**A**) formaldehyde (FA) (10^−8^ M to 10^−5^ M), (**B**) benzene (Bz) (10^−11^ M to 10^−8^ M), or 0.1 % dimethyl sulfoxide (DMSO) (a control) was added for 9 days. The cell proliferation was then evaluated through MTT assay. Values shown are the means ± SD. * mean values were significantly different from control, *p* < 0.05. (Dunnett’s multiple comparison test).

The cell proliferation assay using MTT was conducted to investigate the effects of FA and Bz on proliferation of JEG-3 placenta carcinoma cells. As shown in Figure 1, FA and Bz induced increased cell proliferation within the range of concentrations tested (10^−11^ M to 10^−5^ M) relative to the control. Additionally, FA and Bz were shown to upregulate cell proliferation (Figure 1A,B).

### 3.2. Effects of CS Components on Protein Expression of Cell Cycle Regulatory Genes

**Figure 2:**
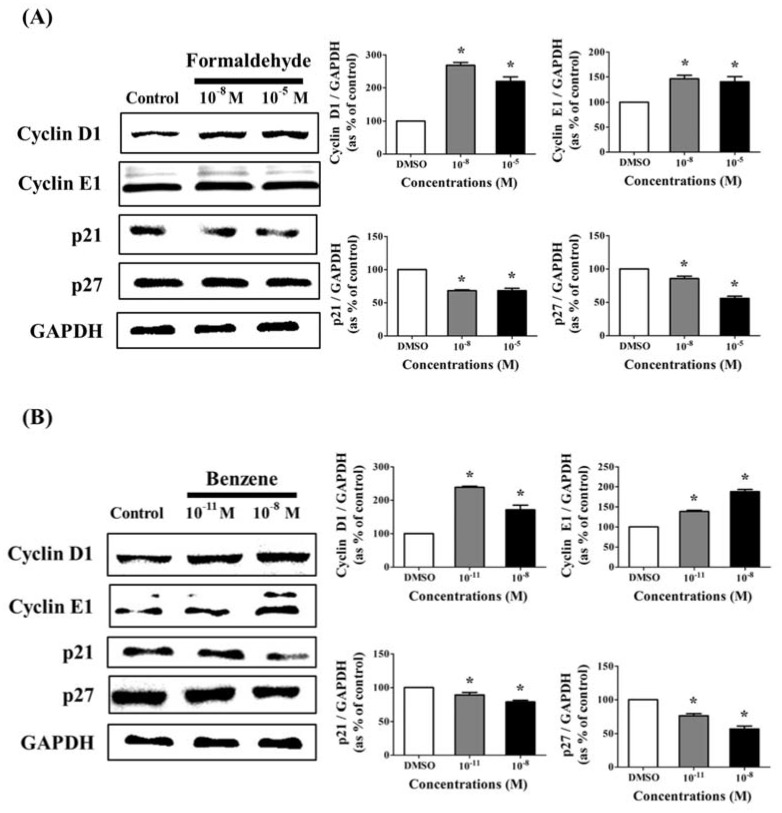
Effect of FA and Bz on protein expression of cell cycle related genes in JEG-3 cells. JEG-3 cells were seeded in 100 mm dishes and treated with medium containing DMSO (control), (**A**) FA (10^−11^ M to 10^−5^ M), or (**B**) Bz (10^−11^ M to 10^−5^ M) for 72 h. After protein extraction, Western blot assay was conducted to conform to the protein expression of cell cycle related genes (cyclin D1, cyclin E1), cell cycle arrest genes (p21 and p27), and housekeeping genes (glyceraldehyde 3-phosphate dehydrogenase (GAPDH)). Quantification of cyclin D1, cyclin E1, p21, and p27 protein was conducted by measuring band densities using a CS analyzer 4 (ATTO, Corp., Japan), and their protein levels were normalized by the band value of GAPDH. Values shown are the means ± SD. * mean values were significantly different from 0.1% DMSO (control), *p* < 0.05. (Dunnett’s multiple comparison test).

Based on the results of the MTT assay, Western blot was performed to evaluate the effects of FA and Bz on the expression of cell cycle related genes such as cyclin D1, cyclin E1, p21, and p27. FA and Bz were observed to increase the protein expressions of cyclin D1 and cyclin E1 and decrease the protein expression of p21 and p27 in a dose dependent manner (Figure 2A,B). FA and Bz affect cancer cell proliferation through induction of the cell cycle progression, which corresponds to the induction of cell proliferation by the treatment of JEG-3 cells with FA and Bz.

### 3.3. FA and Bz Induced Activation of Migration in JEG-3

**Figure 3:**
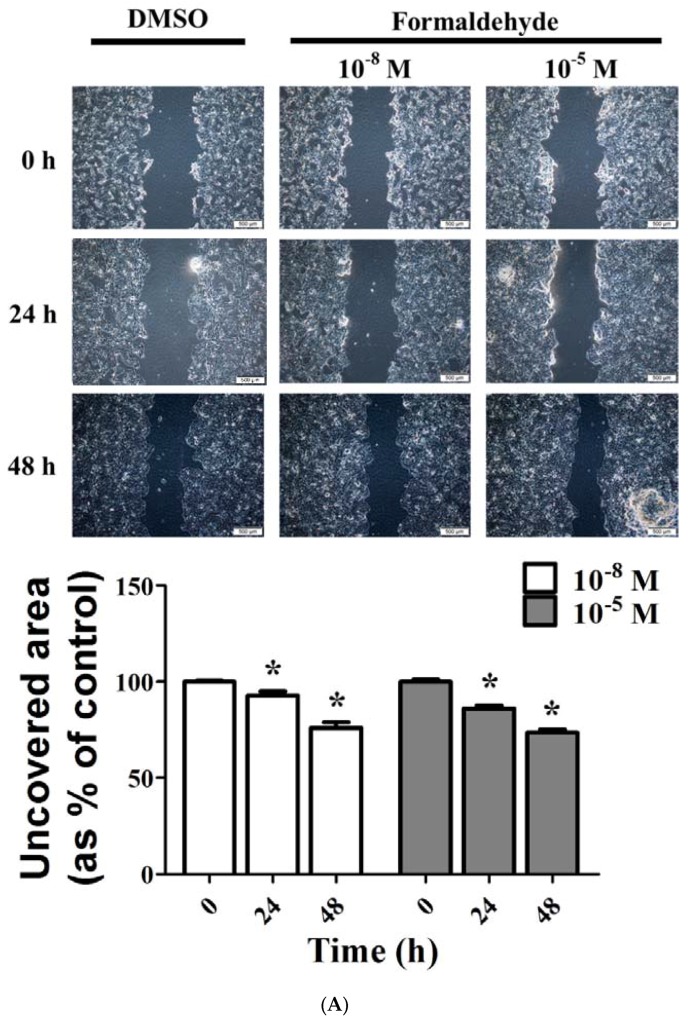

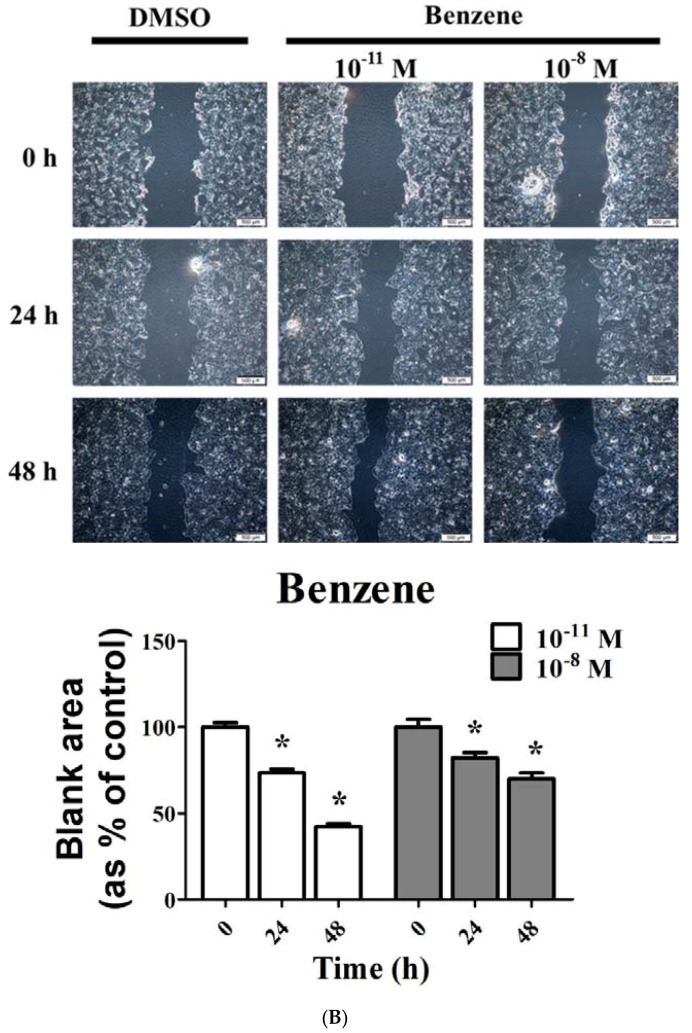
Effect of FA and Bz on migration activity of JEG-3 cells. JEG-3 cells were seeded in 6-well plates at 80% density, after which a region was scratched with the same length and width. Samples were then treated with medium containing 0.1% DMSO (control), (**A**) FA (10^−8^ M and 10^−5^ M), or (**B**) Bz (10^−11^ M and 10^−8^ M) for 48 h. Images shown were taken under a microscope at 40× magnification. Quantification of migration activity was conducted using a Cellsens dimension program. Values shown are the means ± SD. * mean values were significantly different from control, *p* < 0.05. (Dunnett’s multiple comparison test).

A scratch assay was performed to investigate the effects of FA and Bz on the migration of JEG-3 placenta choriocarcinoma cells as seen in Figure 3. After 48 h of treatment with FA and Bz, the mobility of cancer cells through the uncovered area was measured to determine the change. The uncovered area decreased significantly in response to treatment with both FA and Bz relative to DMSO treated cells, and this decrease was shown in a dose-dependent manner (Figure 3A,B). These results indicate that FA and Bz induce the ability of JEG-3 placenta carcinoma cells to migrate.

### 3.4. Effects of CS Components on Protein Expression of EMT Progress Genes

**Figure 4:**
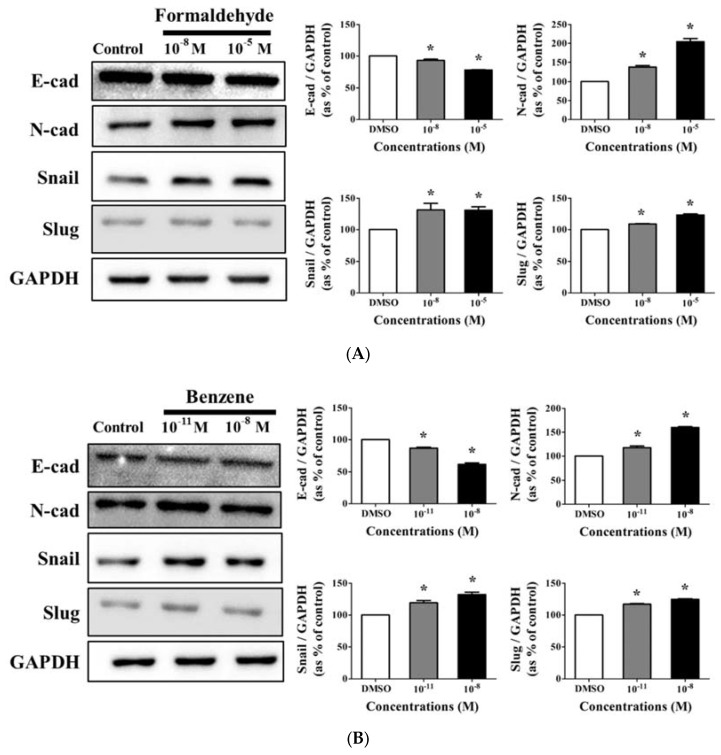
Effect of FA and Bz on protein expression of epithelial mesenchymal transition (EMT) markers in JEG-3. JEG-3 cells were seeded in 100 mm dishes and treated medium with 0.1% DMSO (control), (**A**) FA (10^−8^ M and 10^−5^ M), or (**B**) Bz (10^−11^ M to and 10^−8^ M) for 72 h. After protein extraction, Western blot was conducted to confirm the protein expression of epithelial marker (E-cadherin), mesenchymal markers (N-cadherin, Snail and Slug), and the housekeeping gene (GAPDH). Quantification of E-cadherin, N-cadherin, Snails and Slug protein was conducted by measuring the band densities using a CS analyzer 4 (ATTO, Corp., Japan), and their protein levels were normalized by the band value of GAPDH. Values shown are the means ± SD. * mean values were significantly different from control, *p* < 0.05. (Dunnett’s multiple comparison test).

Western blot assays were conducted using E-cadherin, N-cadherin, Snail, and Slug antibodies, which are considered important EMT markers, to identify the effect of FA and Bz on protein expression [33]. As shown in Figure 4A, FA significantly decreased the protein expression of E-cadherin, but increased that of N-cadherin, Snail, and slug. Bz also significantly decreased the expression of E-cadherin, but increased that of N-cadherin, Snail, and slug (Figure 4B). Moreover, the changes in the expression of EMT markers were dose dependent (Figure 4A,B). These results indicate that FA and Bz induced EMT ability though regulation of the transcription factors Snail and slug, which also are associated with alterations in expression of the cell surface markers E-cadherin and N-cadherin.

### 3.5. FA and Bz Activated ROS Synthesis and Increased Antioxidant Factor

**Figure 5:**
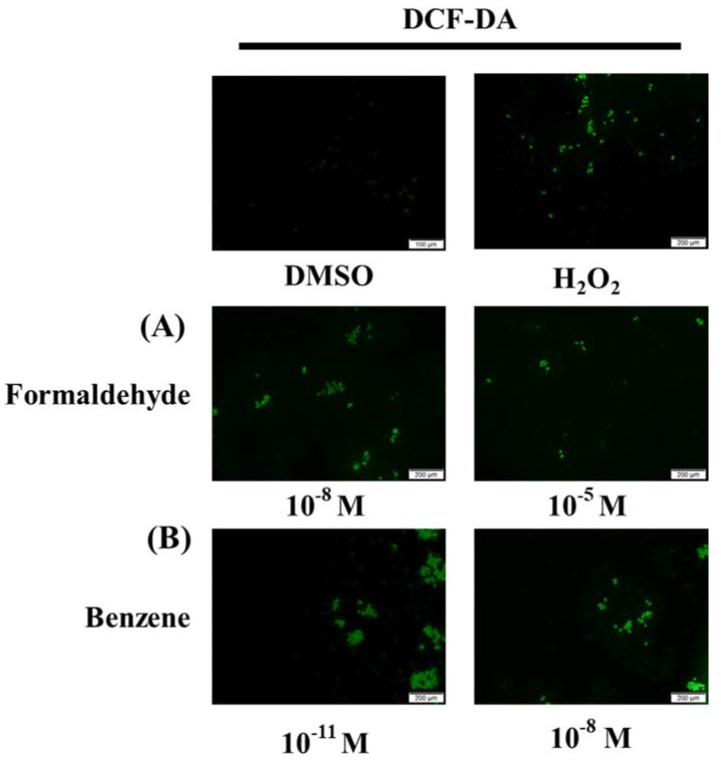
Activation of reactive oxygen species (ROS) by treatment with FA and Bz. JEG-3 cells were seeded at 2 × 10^4^ cell per well in a 96-well plate. After 1 day of incubation, medium treated with (**A**) FA (10^−8^ M and 10^−5^ M), (**B**) Bz (10^−11^ M and 10^−8^ M), or 0.1% DMSO (a control) was added for 72 h, and 3% H_2_O_2_ was treated for 30 min as a positive control to induce ROS. The culture medium was then removed, and each well was treated with 200 µL 20,70-dichlorofluorescein diacetate (DCF-DA) solution for 30 min. To detect ROS activation, fluorescence intensity was measured using an IX70 fluorescence microscope (Olympus, Japan) in a dark room at room temperature.

**Figure 6:**
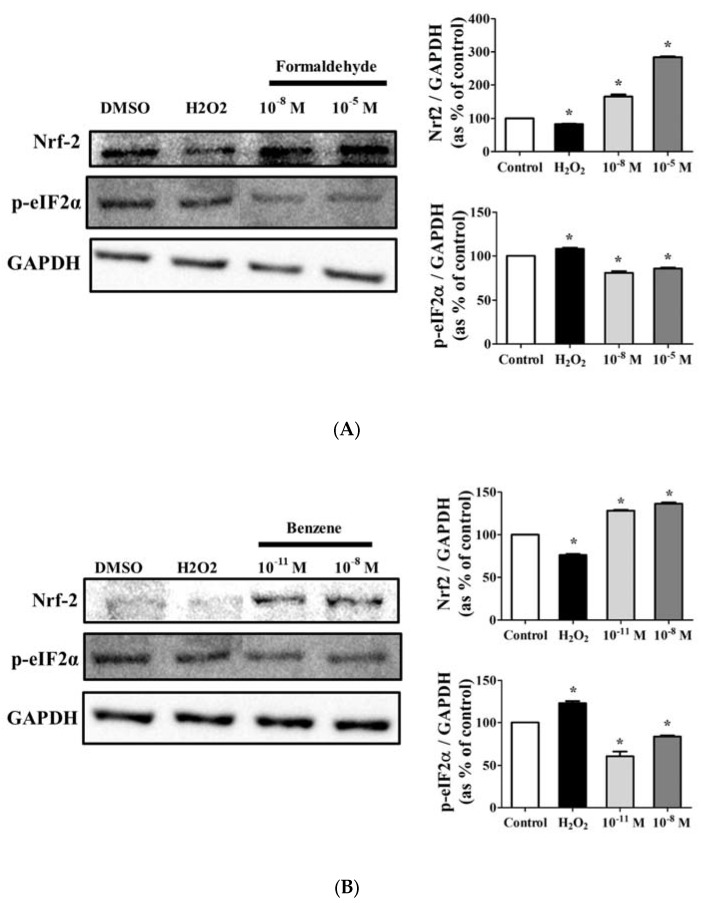
Effect of FA and Bz on protein expression of antioxidant factor nuclear factor erythroid 2 (NFE2)-related factor 2 (Nrf2) and endoplasmic reticulum (ER) stress marker eIF2α in JEG-3. JEG-3 cells were seeded in 100 mm dishes and treated with medium containing 0.1% DMSO (control), (**A**) FA (10^−11^ M and 10^−8^ M), or (**B**) Bz (10^−8^ M and 10^−5^ M) for 72 h. After protein extraction, Western blot was conducted to confirm the protein expression of the antioxidant factor, Nrf2, the ER stress marker, eIF2α, and the housekeeping gene, GAPDH. Quantification of Nrf2 and eIF2α protein was conducted by measuring band densities using a CS analyzer 4 (ATTO, Corp., Japan), and their protein levels were then normalized by the band value of GAPDH. Values shown are the means ± SD. * mean values were significantly different from 0.1% DMSO (control), *p* < 0.05. (Dunnett’s multiple comparison test).

Because up-regulation of cell proliferation and EMT by FA and Bz was observed (Figure 1 and Figure 3), we further evaluated their effects on ROS activation and protein expression of the anti-oxidant factor Nrf2 and the apoptosis related gene p-eIF2α. Fluorescence microscopy analysis using DCF-DA solution revealed that FA and Bz significantly increased activation of ROS in JEG-3 cells compared to that of the control as shown in Figure 5. Western blot assay revealed that FA and Bz increased the protein expression of anti-oxidant factors, but decreased the protein expression of the ER stress-apoptosis related gene p-eIF2α (Figure 6A,B). These findings indicated that FA and Bz may induce cancer cell survival and migration of JEG-3 cells via activation of antioxidation.

## 4. Discussion

A previous study reported that maternal cigarette smoking or exposure to cigarette smoke (CS) alters human placental development via dysregulation of cytotrophoblast proliferation or cellular responses to oxygen [34,35], indicating that cigarette smoke was associate with harmful effects on human placenta or human choriocarcinoma. Accordingly, recent studies have focused on the effects of components of cigarette smoke on the human body.

Nicotine, which is the main constituent among about 5000 toxic components in CS, is a contributing factor to cancer onset, growth, and migration [36]. Moreover, nicotine has potential effects on the endometrium. A previous study showed that nicotine induced endometrial decidualization by decreasing the weight of the uterus after mechanically induced decidualization [37]. Another group also recently observed decreased endometrial proliferation though nitric oxide (NO)-mediated pathways [38]. Moreover, another compound of CS, benzo a pyrene (BaP), is also known to alter cell proliferation of endometrial stroma and epithelial cells via arylhydrocarbon receptor AhR [39].

In the present study, we investigated the effects of FA and Bz, which are the principal components of CS, on cell proliferation and epithelial mesenchymal transition (EMT) of JEG-3 human placenta choriocarcinoma cells. FA significantly increased cell proliferation at a low concentration of 10^−8^ M, while Bz significantly increased cell proliferation at the considerably low concentration of 10^−11^ M. Therefore, we confirmed via Western blot that there were changes in expression of proteins related to the cell cycle such as cyclin D1, cyclin E1, p21, and p27. The cell cycle plays an important role in cells, leading to division, and many proteins are involved in cell cycle checkpoints, including cyclin-dependent kinases (CDKs) and cell cycle arrest genes. Moreover, cyclin D1 and cyclin E1 are known to be major inducers of the cell cycle [40,41]. In a previous study, CS induced survival or migration of cancer cell, while it repressed CDK inhibitors such as p21 and p27. A previous study reported that CS induces the growth of cancer cells by accelerating the G1 phase of the cell cycle [42,43]. In the present study, FA and Bz were found to increase cell proliferation through increased expression of cyclin D1 and E1 in a dose dependent manner. Moreover, these compounds decreased the expression of the cell cycle arrest proteins p21 and p27 in a dose dependent manner.

EMT plays an important role in cancer metastasis and aggravated statuses of cancer patients [44]. Through this process, cells acquire the capacity of motility., which leads to decreased adhesive ability for embryonic development in various tissues or organs [45]. A recent study reported that nicotine and CS induced growth and metastasis [19]. Therefore, we hypothesized that EMT increased in response to FA and Bz in JEG-3. In a scratch assay, two components of CS significantly induced JEG-3 cell migration. Cell migration occurs via regulation of the expression of intracellular protein, which changes the expression of E-cadherin, an epithelial cell marker, and N-cadherin, a mesenchymal marker. Western blot assay revealed that the expression of E-cadherin was reduced in response to treatment with FA and Bz, while the expression of its reverse transition marker, N-cadherin, increased. Moreover, FA and Bz upregulated the EMT associated transcriptional factors, Snail and Slug. Zinc-finger transcription factors play an important role in EMT [46], and Snail not only suppresses epithelial genes, but also promotes mesenchymal gene transcription.

We also focused on the mediation of ROS, antioxidants and ER stress pathways. We previously observed that FA and Bz induced an increase of apoptosis in the human colon cancer cell, SW620 [47]. However, in the present study, FA and Bz induced an increase in cell proliferation and EMT in JEG-3. Therefore, in this study, we focused on changes in the protein level of Nrf2 activated by ROS. ROS are known to induce apoptosis through the C/EBP-homologous protein (CHOP) pathway of ER-stress [48], as well as to activate Nrf2 [49,50]. Nrf2 prevents apoptosis through inactivation of ER stress [51,52]. Although Nrf2 has been known as a transcription factor that regulates the expression of antioxidants and cytoprotective genes under oxidative stress, recent data has revealed that Nrf2 activity is also associated with oncogenic function and survival and metastasis of cancer cells [53], and blockage of Nrf2 inhibited the metastatic abilities such as migration and invasion of esophageal squamous cell carcinoma cells [54]. The overexpression of Nrf2 was identified in a variety of tumors [55], and, especially, it was found that Nrf2 promoted cell proliferation and metastasis by increasing RhoA protein stability and expression in MCF-7 and MDA-MB-231 breast cancer cells [56]. In addition, in pancreatic ductal adenocarcinoma cells, interleukin-6-mediated Stat3 activation induced Nrf2 signaling to promote EMT [57].) Therefore, we assumed that Nrf2 interferes with activation of the ER-stress apoptosis pathway or directly induces increased proliferation of cancer and increased EMT by Nrf2. Activation of intracellular ROS by FA and Bz was observed through DCFH-DA. In the DCFH-DA assay, activation of ROS was significantly increased in JEG-3 cells treated with FA and Bz relative to DMSO, and FA and Bz induced an increase in protein expression of Nrf2. Moreover, the expression of eIF2α was decreased.

## 5. Conclusions

**Figure 7:**
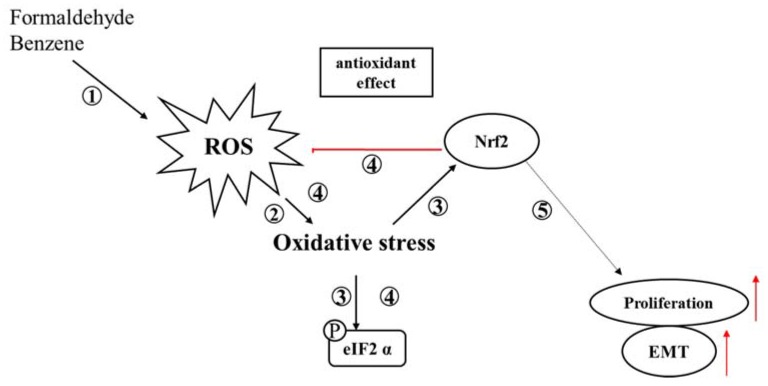
Roles of FA and Bz in the exhibition of cell proliferation and the promotion of EMT or in the prevention of apoptosis. ① FA and Bz activate the intracellular ROS of JEG-3, ② which in turn activates oxidative stress. ③ This activates apoptosis by activating apoptosis-related eIF2, or by activating the antioxidant factor, Nrf2, ④ to regulate the activation of ROS by inducing an antioxidant effect through the increase of Nrf2 which blocks the activation of ROS. However, FA and Bz not only activate ROS, but also increase the activity of Nrf2, which prevents the activation of oxidative stress and apoptosis. ⑤ In addition, FA and Bz induce cell proliferation and EMT, either directly or indirectly through Nrf2. Therefore, FA and Bz inhibit apoptosis through the ROS-Nrf2 pathway, leading to an increase in proliferation and EMT.

The results of the present study showed that FA and Bz induced an increase in proliferation by inducing expression of the cell cycle related proteins, cyclin D1 and cyclin E1, or by reducing expression of the cell cycle arrest proteins, p21 and p27. These chemicals also promoted the EMT process thought up- and down-regulation of EMT related genes such as E-cadherin, N-cadherin, snail, and slug. Moreover, FA and Bz induce the activation of ROS, which activates oxidative stress and increases the expression of Nrf2, an antioxidant factor, thereby blocking ROS activation and preventing normal cells from going to abnormal cell states such as apoptosis as demonstrated in Figure 7. Overall, the results of the present study suggest that FA and Bz may affect cell proliferation and migration of human choriocarcinoma cells JEG-3 through inhibition of ER stress activity and increased activity of Nrf2.
